# Supplementary material for: Targeting MYC dependency in ovarian cancer through inhibition of CDK7 and CDK12/13
Source: eLife. 2018 Nov 13;7:e39030. doi: 10.7554/eLife.39030 (PMC6251623; doi:10.7554/eLife.39030)
Supplement: Supplementary file 1. [file elife-39030-supp1.pdf]

Table S1. Epigenetic and transcriptional inhibitor library (related to Figure 2)

Compound Name	Primary Target	Target category
Olaparib	PARP-1, PARP-2	polyADP ribose polymerase (PARP)
Veliparib	PARP-1, PARP-2	polyADP ribose polymerase (PARP)
Vorinostat	histone deacetylases (HDAC1,2,3,6)	histone deacetylases (HDAC)
Azacitidine	DNMT	DNA methyltransferase (DNMT)
Decitabine	DNMT	DNA methyltransferase (DNMT)
RG-108	DNMT	DNA methyltransferase (DNMT)
Iniparib	PARP1	polyADP ribose polymerase (PARP)
Rucaparib	PARP1	polyADP ribose polymerase (PARP)
JW55	TNKS1/2	polyADP ribose polymerase (PARP)
C646	p300/CREB-binding protein (CBP)	histone acetyl transferases (HAT)
garcinol	p300 and PCAF	histone acetyl transferases (HAT)
Anacardic acid	PCAF and p300	histone acetyl transferases (HAT)
CTB	p300 activator	histone acetyl transferases (HAT)
Belinostat	histone deacetylases (HDAC)	histone deacetylases (HDAC)
Entinostat	histone deacetylases (HDAC1,3)	histone deacetylases (HDAC)
Mocetinostat	histone deacetylases (HDAC, class I,II)	histone deacetylases (HDAC)
Pracinostat	Pan-HDAC	histone deacetylases (HDAC)
MC1568	HDAC4	histone deacetylases (HDAC)
Rocilinostat	HDAC6	histone deacetylases (HDAC)
selisistat	SIRT1	SIRT
AGK2	SIRT2	SIRT
Resveratrol	SIRT1 activator	SIRT
BIX-01294	G9A (EHMT2) and GLP1 (EHMT1) (on H3K9)	histone lysine methyltransferases
UNC0638	G9A (EHMT2) and GLP1 (EHMT1) (on H3K9)	histone lysine methyltransferases
GSK-J1	JMJD3 (on H3K27)	histone lysine demethylase
GSK-J2	JMJD3 (on H3K27)	histone lysine demethylase
GSK-J4	JMJD3 (on H3K27)	histone lysine demethylase

Daminozide	KDM2/7	histone lysine demethylase
methylstat	KDM4A/JMJD2A	histone lysine demethylase
tranylcypromine	LSD1 and LSD2	histone lysine demethylase
PFI-1	BET (BRD2 and BRD4)	Bromodomain
(+)-JQ1	BET (BRD2, 3, 4, and T)	Bromodomain
(-)-JQ1	BET (BRD2, 3, 4, and T)	Bromodomain
GSK 525762A	BET (BRD2/3/4 specific)	Bromodomain
GSK1210151A	BET (BRD4 and BRD3)	Bromodomain
Ischemin	CREB-binding protein	Bromodomain
UNC669	L3MBTL1	MBT
UNC1215	L3MBTL3	MBT
IOX2	PHD finger	PHD finger
Epigallocatechin gallate	Telomerase	Telomerase
CX-5461	RNA polymerase I	RNA polymerase
THZ1	CDK7, CDK12, CDK13	transcriptional CDK
