# Supplementary material for: Targeting MYC dependency in ovarian cancer through inhibition of CDK7 and CDK12/13
Source: eLife. 2018 Nov 13;7:e39030. doi: 10.7554/eLife.39030 (PMC6251623; doi:10.7554/eLife.39030)
Supplement: Supplementary file 2. [file elife-39030-supp2.pdf]

**Table S2. Normalized MYC expression of compound screen (related to Figure 2)**

| | Ku | Cov |
|---|---|---|
| DMSO | 1 | 1 |
| THZ1 | 0.122 | 0.222 |
| Olaparib | 1.428 | 1.02 |
| Veliparib | 1.059 | 1.01 |
| Vorinostat | 0.957 | 0.852 |
| Azacitidine | 1.322 | 0.989 |
| Decitabine | 1.16 | 0.811 |
| RG108 | 1.329 | 1.034 |
| Iniparib | 1.145 | 1.068 |
| Rucaparib | 0.905 | 1.036 |
| JW55 | 0.599 | 0.834 |
| DMSO | 1 | 1 |
| THZ1 | 0.076 | 0.209 |
| EX527 | 0.655 | 1.208 |
| AGK2 | 0.703 | 1.367 |
| Resveratrol | 0.721 | 1.501 |
| BIX01294 | 0.86 | 1.177 |
| UNC0638 | 1.186 | 1.326 |
| GSK-J1 | 0.953 | 1.408 |
| GSK-J2 | 0.931 | 1.266 |
| GSK-J4 | 0.871 | 1.079 |
| Daminozide | 0.975 | 0.871 |
| Methylstat | 0.604 | 1.051 |
| Tranylcypromine | 1.129 | 1.207 |

| | Ku | Cov |
|---|---|---|
| DMSO | 1 | 1 |
| THZ1 | 0.099 | 0.223 |
| C646 | 0.964 | 0.899 |
| Garcinol | 0.786 | 0.958 |
| Anacardic acid | 0.996 | 1.088 |
| CTB | 0.811 | 0.98 |
| Belinostat | 0.603 | 0.779 |
| MS275 | 0.742 | 1.032 |
| Mocetinostat | 0.911 | 0.828 |
| SB939 | 0.722 | 0.722 |
| MC1568 | 0.914 | 0.948 |
| Rocilinostat | 0.843 | 0.919 |
| DMSO | 1 | 1 |
| THZ1 | 0.096 | 0.212 |
| PFI-1 | 0.845 | 1.091 |
| JQ1 | 0.794 | 0.74 |
| (-)JQ1 | 1.115 | 1.342 |
| I-BET | 0.825 | 0.96 |
| I-BET151 | 0.871 | 0.787 |
| Ischemin | 0.946 | 1.315 |
| UNC669 | 1.253 | 1.235 |
| UNC1215 | 1.183 | 1.196 |
| IOX2 | 0.949 | 1.331 |
| EGCG | 1.011 | 1.112 |
| CX5461 | 0.958 | 0.934 |
